# Umifenovir Epigenetically Targets the *IL-10* Pathway in Therapy against Coxsackievirus B4 Infection

**DOI:** 10.1128/spectrum.04248-22

**Published:** 2022-12-21

**Authors:** Shilin Zhang, Xiao Xue, Sennan Qiao, Lin Jia, Xue Wen, Yichen Wang, Cong Wang, Hongrui Li, Jiuwei Cui

**Affiliations:** a Key Laboratory of Organ Regeneration and Transplantation of Ministry of Education, Cancer Center, First Hospital of Jilin University, Changchun, Jilin, People’s Republic of China; b Department of Clinical Laboratory, First Hospital of Jilin University, Changchun, Jilin, People’s Republic of China; c Institute of Frontier Medical Science of Jilin University, Changchun, Jilin, People’s Republic of China; Institute of Microbiology, Chinese Academy of Sciences

**Keywords:** CVB4 infection, *IL10*, epigenetics, chromatin remodeling, histone modification

## Abstract

Umifenovir, a broad-spectrum nonnucleoside antiviral drug, has a promising efficacy against coxsackievirus B4 (CVB4) infection, but its mechanism remains unclear. CVB4 is a common human single-stranded RNA virus that belongs to the *Picornaviridae* family and the *Enterovirus* genus. *Enterovirus* can cause severe diseases, such as meningitis, myocarditis, pancreatitis, insulin-dependent diabetes, and several other diseases, in both adults and children. We have previously demonstrated the critical role of interleukin 10 (IL-10) in promoting CVB4 infection and the downregulation of IL-10 by umifenovir. To further explore the underlying mechanisms of umifenovir, we characterized the epigenetic regulation of IL-10 in *IL-10* knockout RAW264.7 cells and a BALB/c mouse splenocyte model. Mechanistically, we found that umifenovir inhibited CVB4-activated IL-10 by enhancing the methylation level of the repressive histones H3K9me3 and H3K27me3 while reducing the acetylation level of the activating histone H3K9ac in the promoter region of the *IL-10* gene. Furthermore, using a chromosome conformation capture approach, we discovered that CVB4 infection activated the *IL-10* gene by forming an intrachromosomal interaction between the *IL-10* gene promoter and an intronic enhancer of the downstream *MK2* (mitogen-activated protein kinase [MAPK]-activated protein kinase 2 [*MAPKAPK2*]) gene, a critical component of the p38-MAPK signaling pathway, which is required for *IL-10* gene expression. However, umifenovir treatment abolished this spatial conformation and chromatin interaction, thus reducing the continuous expression of IL-10 and subsequent CVB4 replication. In conclusion, this study reveals a novel epigenetic mechanism by which umifenovir controls CVB4 infections, thus laying a theoretical foundation for therapeutic use of umifenovir.

**IMPORTANCE** Viral infections are major threats to human health because of their strong association with a variety of inflammation-related diseases, especially cancer. Many antiviral drugs are performing poorly in treating viral infections. This is probably due to the immunosuppressive effect of highly expressed IL-10, which is caused by viral infection. Umifenovir is a broad-spectrum antiviral drug. Our recent studies showed that umifenovir has a significant inhibitory effect on CVB4 infection and can reduce IL-10 expression caused by CVB4. However, another antiviral drug, rupintrivir, showed good antiviral activity but had no effect on the expression of IL-10. This suggests that the regulation of IL-10 expression is a key part of the antiviral mechanism of umifenovir. Therefore, due to the dual function of the inhibition of CVB4 replication and the regulation of immune antiviral pathway, the mechanism of umifenovir is of great value to study.

## INTRODUCTION

The COVID-19 pandemic has brought unprecedented challenges to human society. Viral infections remain major threats to human health. Many viruses, if not promptly removed, can lead to a variety of inflammation-related diseases (reviewed in reference 1), including cardiovascular diseases, neurodegeneration, allergic diseases, cancer (2, 3), and autoimmune diseases. Coxsackievirus B4 (CVB4) is a common human single-stranded RNA virus belonging to the *Enterovirus* genus in the family *Picornaviridae*. *Enterovirus* is the main pathogen that causes viral myocarditis, insulin-dependent diabetes, and several other diseases in adults and children. Umifenovir has a significant inhibitory effect on CVB4 infection (4). It is a broad-spectrum antiviral drug and has the advantages of high antiviral activity, low toxicity, and low susceptibility to drug resistance. In several recent studies, umifenovir was also reported to be effective against COVID-19 (5, 6). However, the antiviral mechanism of umifenovir still requires further investigation.

Several studies have documented that umifenovir can prevent viruses from entering cells and inhibit viral replication and budding by interacting with the cell membrane, viruses, or cellular proteins (reviewed in reference 7). Our recent studies showed that umifenovir can reduce the expression level of interleukin 10 (IL-10) and the CVB4 viral load in a mouse model of viral myocarditis and alleviate pathological damage in the myocardium (4). We confirmed that umifenovir blocks the process of nuclear translocation of p38 into the nucleus and the p38-MK2 complex out of the nucleus, thus blocking the biological function of the p38–mitogen-activated protein kinase (MAPK) signaling pathway and inhibiting IL-10 expression caused by CVB4 infection. We also evaluated the effect of umifenovir on IL-10 secretion stimulated by lipopolysaccharide (LPS) and found that umifenovir could significantly inhibit IL-10 secretion in a dose-dependent manner. This illustrated that umifenovir could inhibit IL-10 expression caused by various stimuli. However, we have already demonstrated that another antiviral drug, rupintrivir, a 3C protease inhibitor, has good anti-CVB4 activity but no effect on the expression of IL-10. This result suggested that in addition to its interactions with cell membranes and proteins, the antiviral activity of umifenovir is strongly associated with the downregulation of IL-10. Therefore, the distinct antiviral mechanism of umifenovir has high value for research.

In light of the wide spectrum of anti-inflammatory properties, IL-10 plays an important role in a variety of microbial infections and possesses potential therapeutic effects against several autoimmune diseases and even cancer (reviewed in references 8 to 10). Established evidence indicates that a variety of epigenetic mechanisms are involved in the regulation of IL-10 (11). The field of epigenetics deals with various hereditary changes that influence gene expression without affecting DNA sequences (12) and play an important role in human physiology and diseases (13). Studies have shown that complex epigenetic mechanisms contribute to the invasion, transmission, and persistent infection process of different viruses (reviewed in references 14 and 15). In this study, we explored the epigenetic mechanism by which umifenovir downregulated the expression of IL-10, thus inhibiting CVB4 infections and laying a theoretical foundation for novel therapeutic utilization of umifenovir.

## RESULTS

### Construction and identification of *IL-10* knockout RAW264.7 cells.

**FIG 1:**
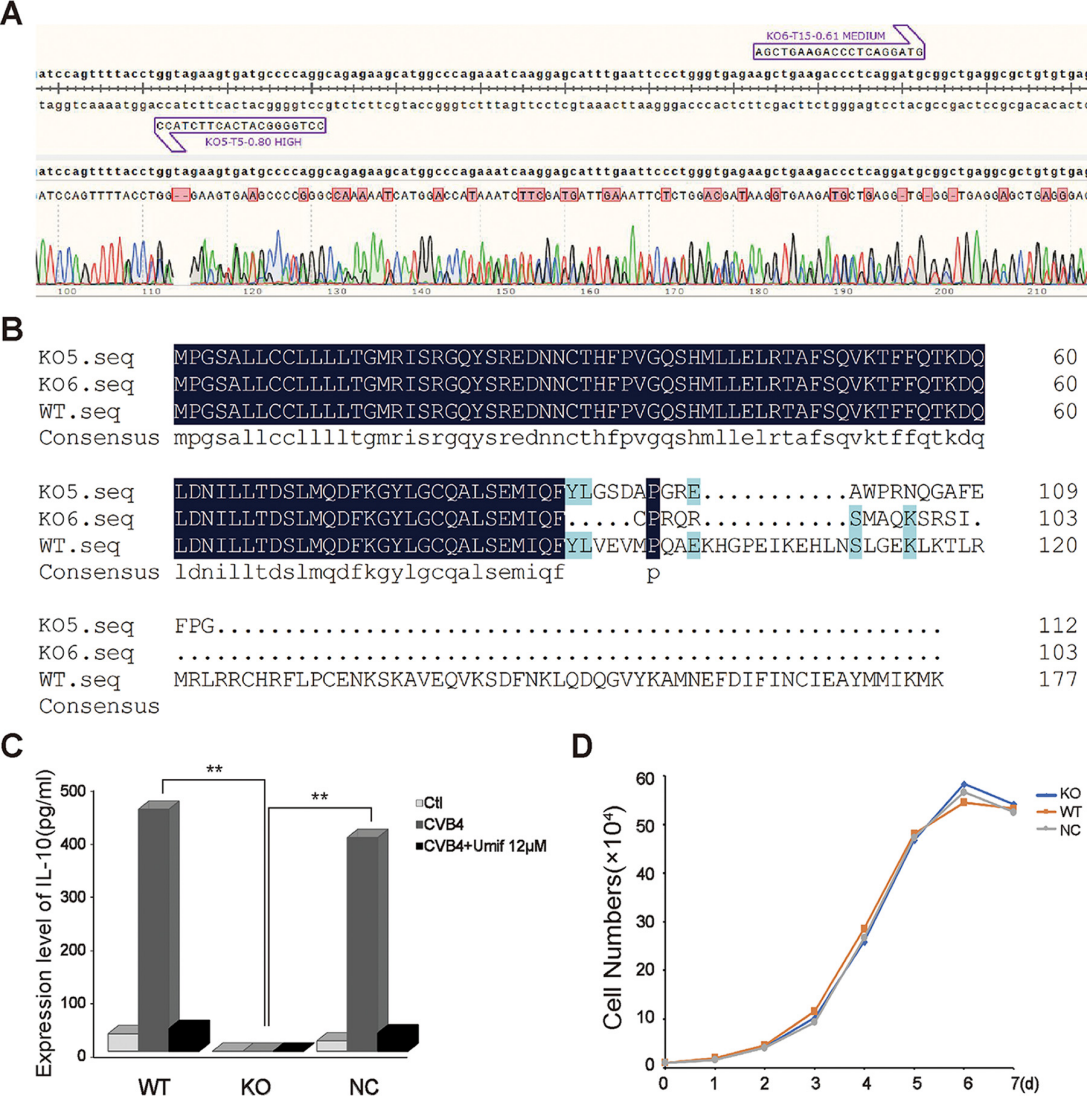
Construction and identification of IL-10 knockout RAW264.7 cells. (A) Sequencing results of *IL-10* knockout RAW2647 cell I-C5# clone. KO5 and KO6 are knockout sites of I-C5#. (B) Prediction of amino acid sequence of RAW2647/KO cells. (C) Identification of IL-10 expression in RAW264.7/KO, RAW264.7/WT, and RAW264.7/NC cells 24 h after CVB4 (1.4 × 10^6^ TCID_50_) infection (ELISA). WT, wild-type RAW264.7 cells; KO, *IL-10* knockout RAW264.7 cells; NC, negative-control RAW264.7 cells that were transfected with empty lenti-CRISPR v2 plasmid. **, *P* < 0.01. (D) Cell growth curves of RAW264.7/KO, RAW264.7/WT, and RAW264.7/NC cells.

To investigate the associations between CVB4 infection and the aberrant activation of the IL-10 pathway, we first established a CVB4 infection model in *IL-10* knockout (KO) RAW264.7 macrophages using the CRISPR/Cas9 system. Single guide RNAs (sgRNAs; KO1 to KO6) designed from the first two exons of *IL-10* were cloned into the lenti-CRISPR guide RNA (gRNA) vector, packaged as lentiviruses, and transfected into RAW264.7 cells. After screening and sequencing, we finally identified an *IL-10* knockout RAW264.7 cell clone, I-C5#. Direct DNA sequencing confirmed that I-C5# carried a heterozygous combination at both KO5 and KO6 knockout sites (Fig. 1A). Although the *IL-10* gene has only one transcript (GenBank accession number NM_010548.2), clone I-C5# had two knockout situations. As a result, two translations of the coding DNA sequence (CDS) region were predicted (Fig. 1B), and both knockouts disrupted the integrity of the CDS domain, resulting in early termination of protein translation.

The knockdown efficacy of IL-10 was validated using enzyme-linked immunosorbent assay (ELISA) (Fig. 1C). The results showed that RAW264.7/KO cells produced barely any IL-10 after 24 h of CVB4 infection, while both RAW264.7/WT (wild type) and RAW264.7/NC (negative control) cells produced plentiful amounts of IL-10 in cell supernatants. Then we evaluated the effect of *IL-10* knockout on cellular growth in RAW264.7 cells through the observation of cellular morphology and the plotting of the cell growth curve (Fig. 1D). RAW264.7/KO cells grew at approximately the same rate as RAW264.7/WT and RAW264.7/NC cells. This illustrated that knockout of IL-10 did not affect the growth of cells. Thus, the *IL-10* knockout RAW264.7 cell clone was successfully constructed and could be used for subsequent experiments.

### Knockout of *IL-10* inhibits CVB4 replication.

**FIG 2:**
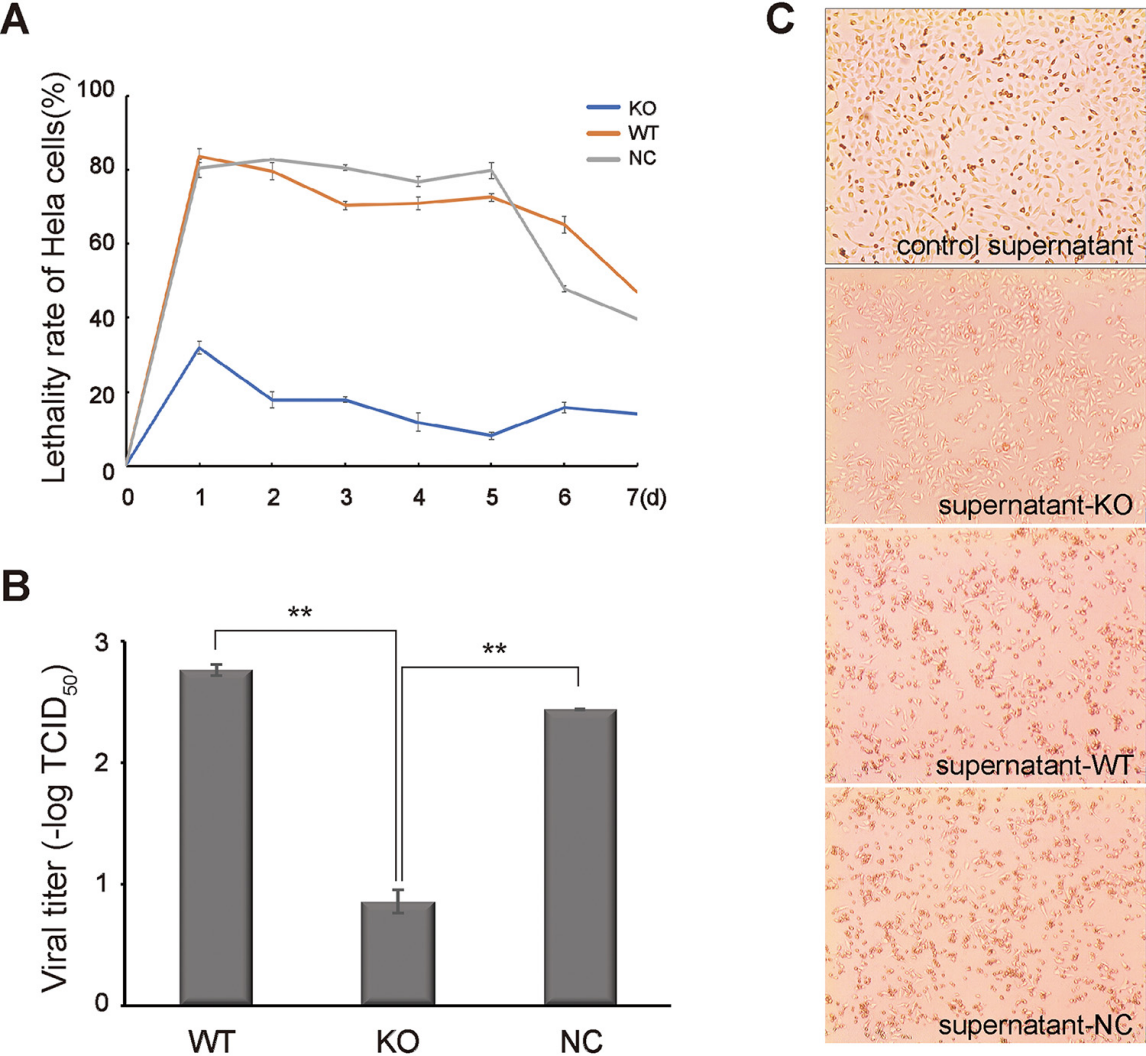
Knockout of IL-10 inhibits CVB4 replication. (A) Lethality rates of HeLa cells which were incubated with viral supernatants of RAW264.7/KO, RAW264.7/WT, and RAW264.7/NC cells. The viral supernatants were collected from CVB4 (1.4 × 10^6^ TCID_50_)-infected RAW264.7/WT, RAW264.7/KO, and RAW264.7/NC cells every day for 7 days. (B) The viral titers of the supernatants (24 h postinfection) of RAW264.7/WT, RAW264.7/KO, and RAW264.7/NC cells were detected using HeLa cells after 48 h in culture. **, *P* < 0.01. (C) The cytopathic effect (CPE) of HeLa cells was observed after 48 h of incubation with control supernatant and viral supernatants (collected at 24 h postinfection) of RAW264.7/KO, RAW264.7/WT, and RAW264.7/NC cells.

To validate the vital importance of IL-10 expression in CVB4 infection, we then assessed the replication of CVB4 in *IL-10* knockout RAW264.7 cells. We found that CVB4 was capable of completing the entire process of adsorption, penetration, synthesis, and release in RAW264.7 cells without LPS treatment, even though RAW264.7 cells are not as susceptible to CVB4 as HeLa cells. Supernatants of CVB4-infected RAW264.7/WT, RAW264.7/KO, and RAW264.7/NC cells were collected every day for 7 days. Supernatant-KO, supernatant-NC, supernatant-WT, and control supernatant, which were diluted in a 10-fold dilution series, were used to incubate HeLa cells. After 48 h in culture, the cytopathic effect (CPE) was observed under a microscope (Fig. 2C), the viral titer were calculated (Fig. 2B), and the lethality rates of infected HeLa cells (Fig. 2A) were assessed using the CCK-8 assay.

The lethality rates of the WT and NC groups reached 83.7% and 80.3% on the first day, while that of the KO group was only 31.9%. The differences are significant in all 7 days’ results. Viral titers were detected after 72 h of incubation, and the results showed that the viral titers in supernatant-WT and -NC were significantly higher than that in supernatant-KO (almost 3-fold). The photographs of HeLa cells more clearly reflected that cells in the KO group had better survival than those in the WT and NC groups. These data indicated that IL-10 deficiency significantly suppressed the replication of CVB4, and they further demonstrated the critical role of IL-10 in viral infections.

### Umifenovir inhibits CVB4 replication by targeting IL-10.

**FIG 3:**
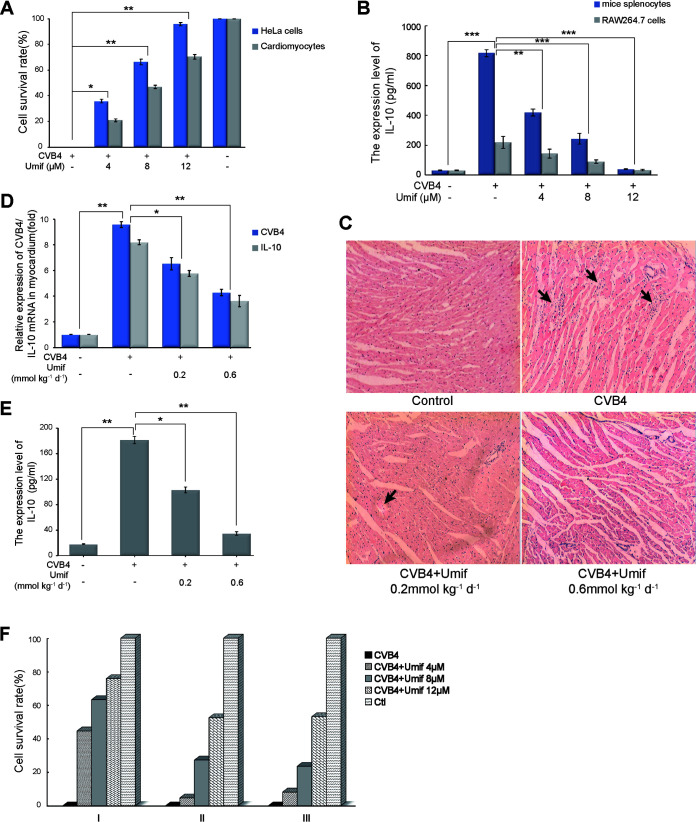
Umifenovir inhibits both CVB4 replication and IL-10 expression *in vitro*, *in vivo*, and *ex vivo*. (A) Anti-CVB4 activity of umifenovir. HeLa cells and cardiomyocytes were infected with CVB4 (1.4 × 10^6^ TCID_50_) and treated with umifenovir (4, 8, and 12 μM). (B) Downregulation of IL-10 by umifenovir (ELISA). Mouse splenocytes and RAW264.7 cells were infected with CVB4 (1.4 × 10^6^ TCID_50_) and treated with umifenovir (4, 8, and 12 μM). (C) Myocardial tissue sections of mice were stained with H&E after CVB4 infection (2.8 × 10^3^ TCID_50_) and umifenovir treatment (0.2 and 0.6 mmol kg^−1^ of body weight day^−1^). Black arrows indicated the necrotic areas (magnification, ×200). (D) Expression level of CVB4 mRNA and IL-10 mRNA in myocardium (RT-PCR). (E) Expression level of IL-10 in mouse serum by ELISA. (F) Direct-acting and host-targeting antiviral mechanisms of umifenovir. Group I, coincubated CVB4 and umifenovir for 2 h, added to HeLa cells for 2 h, discarded the supernatant, and added different dose of umifenovir; group II, added CVB4 to HeLa cells for 2 h, discarded the supernatant, and added different dose of umifenovir; group III, added umifenovir to HeLa cells for 2 h, discarded the supernatant, added CVB4 for 2 h, discarded the supernatant again, and added different dose of umifenovir. Subgroups, viral control (2.8 × 10^3^ TCID50), 4 μM umifenovir, 8 μM umifenovir, 12 μM umifenovir, and normal control. *, *P* < 0.1; **, *P* < 0.01; ***, *P* < 0.001.

As a broad-spectrum antiviral drug, umifenovir has been demonstrated as an effective therapy to inhibit CVB4 replication. To elucidate the mechanism of umifenovir, we first established a CVB4 infection model in mouse cardiomyocytes and HeLa cells. CVB4 infection caused significant cell death in both types of cells, and umifenovir treatment effectively increased the cell survival in a dose-dependent manner (Fig. 3A).

We further examined the role of umifenovir in IL-10 expression in both *in vivo* and *ex vivo* models. In the *ex vivo* model, CVB4 infection in both mouse splenocytes and RAW264.7 cells caused the secretion of substantial amounts of IL-10. However, intervention with umifenovir led to a dose-dependent decrease in IL-10 secretion (Fig. 3B).

In the *in vivo* model, we examined viral myocarditis in CVB4-infected mice. In hematoxylin and eosin (H&E)-stained sections of mouse myocardium, there were large areas of necrosis with massive infiltration of inflammatory cells in the CVB4 infection group (Fig. 3C). However, in the umifenovir treatment group, the necrotic area of myocardial tissue was reduced significantly with increased doses of umifenovir. These data show that umifenovir effectively inhibited the disruptive effect of CVB4 on mouse myocardium.

Further analyses by real-time PCR (RT-PCR) analyses also supported the notion that the viral load in myocardium was positively correlated with the expression level of IL-10 and that umifenovir downregulated IL-10 in a dose-dependent manner (Fig. 3D). This was also supported by ELISA data (Fig. 3E). These data suggest that umifenovir has both an anti-CVB4 effect and the ability to downregulate IL-10 expression, and the antiviral role may be achieved by the downregulation of IL-10.

Antiviral drugs are currently divided into two classes: direct-acting antivirals (DAAs) and host-targeting antivirals (HTAs). To clarify the antiviral mode of umifenovir, we examined the direct-acting and host-targeting antiviral activities of umifenovir. The survival rate of group I was significantly higher than those of groups II and III (Fig. 3F), suggesting that umifenovir has a direct killing effect on CVB4. The antiviral effects of umifenovir in groups II and III had no significant differences. This illustrated that umifenovir can inhibit CVB4 replication following viral adsorption. Therefore, umifenovir is both a DAA and an HTA in CVB4 infection.

### Umifenovir downregulates IL-10 by altering histone epigenotypes in the gene promoter.

**FIG 4:**
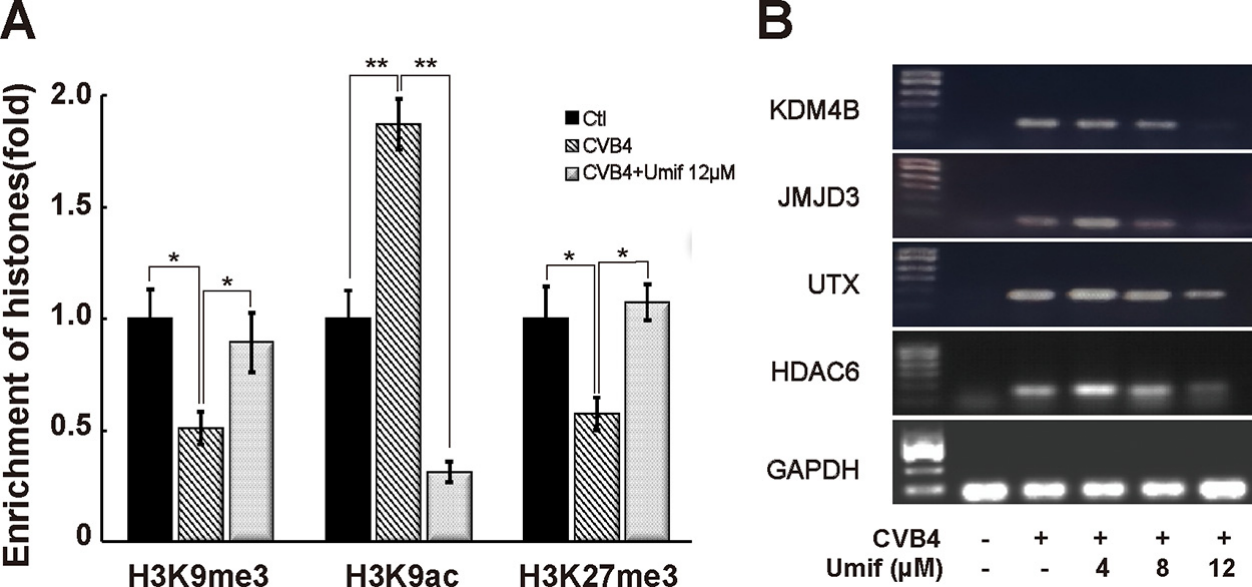
Umifenovir regulates histone epigenotypes in the *IL-10* gene promoter. (A) Alteration of histone epigenotypes in *IL-10* promoter region by umifenovir (ChIP and qPCR). Mouse splenocytes were collected 48h post CVB4 (1.4 × 10^6^ TCID_50_) infection and treated with umifenovir (12 μM). *, *P* < 0.1; **, *P* < 0.01. (B) Expression of histone-modifying enzymes of H3K9me3, H3K9ac, and H3K27me3.

Due to the dual effects of anti-CVB4 and downregulation of IL-10, the investigation of the mechanism of umifenovir is extremely important. We speculated that umifenovir could inhibit IL-10 expression through an epigenetic mechanism, therefore suppressing CVB4 replication. We used chromatin immunoprecipitation (ChIP) and RT-quantitative PCR (RT-qPCR) to examine histone modifications in the promoter region of the *IL-10* gene in mouse splenocytes (normal control, CVB4-infected, and umifenovir treatment groups). The results showed that compared with that in the normal control group, the methylation levels of histones H3K9me3 and H3K27me3 in *IL-10* promoter region decreased significantly in the CVB4-infected group (Fig. 4A). The acetylation level of H3K9ac was also increased significantly. However, in the umifenovir treatment group, the methylation levels of H3K9me3 and H3K27me3 were significantly increased, while the acetylation level of H3K9ac was significantly reduced, to the level seen in the normal control group. These data suggest that umifenovir could inhibit IL-10 expression by elevating the repressive markers H3K9me3 and H3K27me3 and inhibiting the active marker histone H3K9ac in the promoter region of the *IL-10* gene.

To further examine how umifenovir regulates histone modification, we examined the expression of histone modification enzymes, including the demethyltransferases JMJD3 and UTX of H3K27me3, the histone demethyltransferase KDM4B of H3K9me3, and the H3K9ac-specific acetylase HDAC6. As shown in Fig. 4B, CVB4 infection significantly increased the expression of JMJD3, UTX, KDM4B, and HDAC6. However, the treatment with increasing doses of umifenovir, significantly reduced the expression levels of these four histone-modifying enzymes. Thus, we concluded that umifenovir may modify histones H3K27me3, H3K9Me3, and H3K9ac by inhibiting the expression of JMJD3, UTX, KDM4B, and HDAC6, leading to the downregulation of IL-10.

### Umifenovir coordinates a three-dimensional chromatin conformation in the *IL-10* locus.

**FIG 5:**
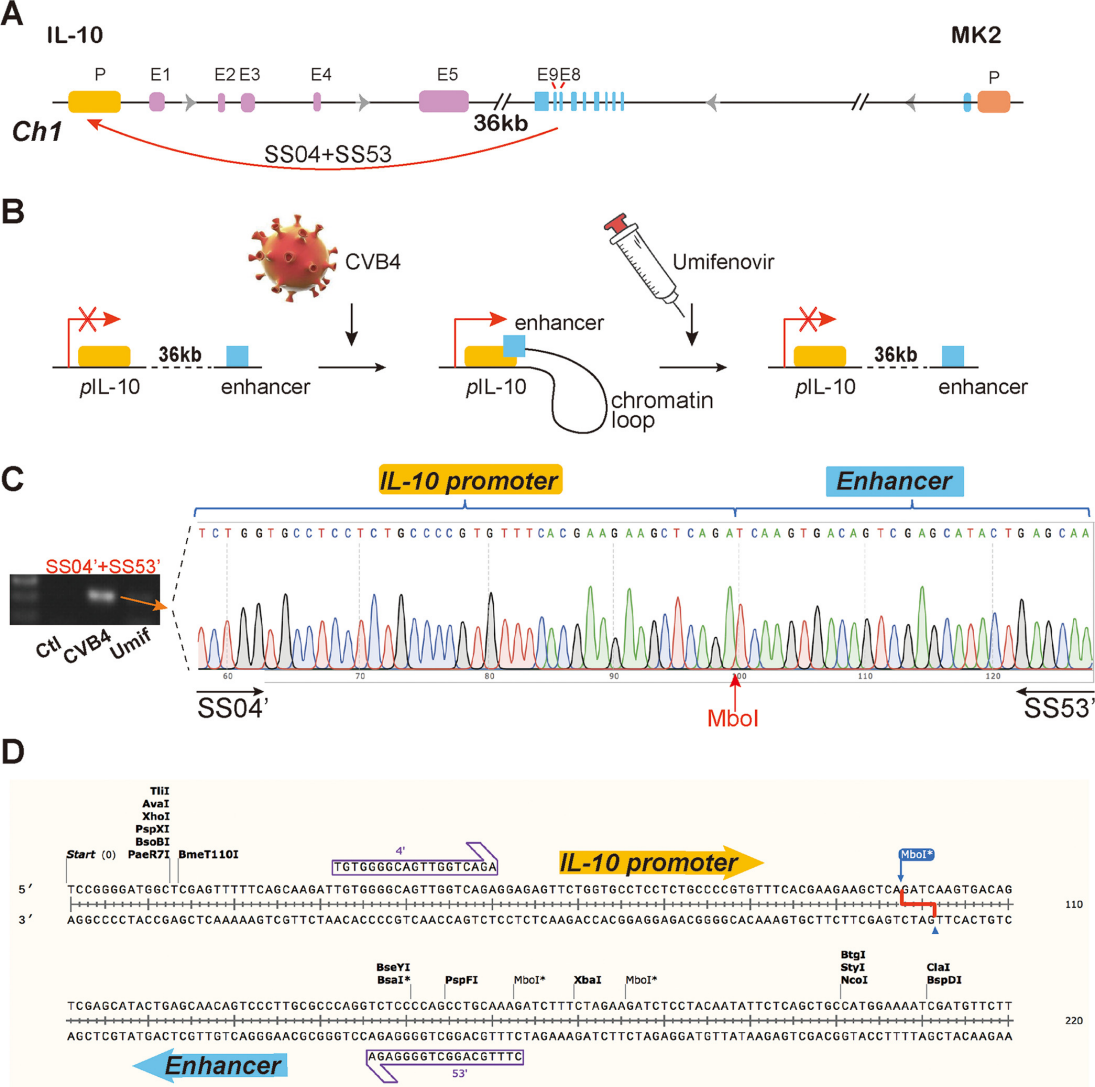
Umifenovir coordinates a three-dimensional chromatin conformation in *IL-10* locus. (A) Positions of the *MK2* and *IL-10* genes and the chromatin interacting sites. E1 to E9, exons; p, promoter. Arrow, *IL-10-MK2* chromatin interaction. (B) Schematic diagram of the formation and destruction of the chromatin loop in the *IL-10* promoter region. (C and D) Sequencing results of 3C-PCR. SS04, SS53, SS04′, and SS53′ are 3C primers of the *IL-10* promoter and *MK2* enhancer for nested PCR. Mouse splenocytes were collected 48 h after CVB4 (1.4 × 10^6^ TCID_50_) infection and treated with umifenovir (12 μM). Red arrow, ligation site (MboI) of 3C products.

Alterations of epigenotypes are closely associated with the three-dimensional chromatin conformation, particularly the intrachromosomal and interchromosomal promoter-enhancer interactions. We thus used a chromatin conformation capture (3C) approach to examine if the altered epigenotypes following CVB4 infection are related to local chromatin structure. The *IL-10* gene locus is located 36 kb upstream of the *MK2* (MAPK-activated protein kinase 2 [*MAPKAPK2*]) gene, which is a critical component of the p38-MAPK signaling pathway (Fig. 5A). *MK2* is required for IL-10 production and is closely related to the expression of *IL-10*. Therefore, we hypothesized that in the context of CVB4 infection, the *MK2* gene may interact with *IL-10* in the same chromatin conformation hub, causing abnormal expression of IL-10.

To test this hypothesis, we designed 3C primers (Table 1) at the promoter region of *IL-10* and used the 3C assay to map its interaction with *MK2*. Using this approach, we identified an *IL-10*-*MK2* chromatin interaction that is associated with CVB4 infection (Fig. 5B). In the 3C products of the CVB4-infected mouse splenocytes, we found an intrachromatin interaction that links the *IL-10* gene promoter to an enhancer in the *MK2* intron 8 region, about 43,985 bp apart, by PCR and electrophoresis. However, the use of umifenovir abolished this chromatin loop.

**TABLE 1:** 3C primers

Primer	Sequence
*IL-10* promoter	
SS01	AGCCACTGCATCAGATAAGACG
SS02	GCAATGCTACACGTCCTGTTGA
SS03	GGCTGGGACATTGTAAAACAGG
SS04	TTGAGGATATGTGTGCTGTGGG
SS05	GCATTGTCTCTCCCATGCTCA
SS06	GGACCTCCATACCAGTTCCAG
SS07	CTCATCTGAAAGAAGACCGGGC
SS08	TGCCCTGGCAACTGTGGAA
SS09	GGGTTGTGAAGAGCTCATTCTG
SS10	GAGGTTGGGTGTGACTTACTGG
SS11	GCATACCTTGTTCGATTGGAGAA
SS12	CCTGTTTCTCCTATACCTTCTCCC
MK2 enhancer	
SS13	ATGTGACTTCGCTCTCCTCG
SS14	GTACCAGCACTAGGCCAAGC
SS15	CTTGGGAAAGCAGATGTTTAGATGC
SS16	CCTCGGTCAAGTGATGGCAT
SS17	CCACATCCCTCCTTGTAAATGG
SS18	TAACCCCTAAAAACAGGCCCATCTC
SS19	TGGGTAGGATGTCTCTTAGCTGC
SS20	CTCCACAGAGGCAGAGGACTT
SS21	GGGCATAGTAAAGCCCTTGGC
SS22	AGAAGACAGTGAGGGTCCCT
SS23	CTGCGTCTGGGCTCATGT
SS24	CAGCTGGACTCCTGCCCA
SS25	GCAACCACTCATCTGTCTAGCTGA
SS26	TCCCTGCAGTGCGCTAGCTA
SS27	ATGTGTGCCCCTGTCTGT
SS28	ACTCCACGTCCAGACCTATGAT
SS29	AGATTGACTAAGGATTGTGGAGGTT
SS30	ACAAGAAAGGGCTGAAGAGATGAT
SS31	AGCTGGTTTTAGGCATTGATAGCC
SS32	CATGTGATGGACTTTGCTGAGACA
SS33	TTGACCTGCTTGCTGCCACT
SS34	CCACGGTCTGCACTGCACA
SS35	CCAAAATAGCACCACCATGGG
SS36	GCAAGGAAGCAATCCGCAAT
SS37	GGAATTCATATTCAGGTGGGGAATC
SS38	CGGCAGAATATCTTTCCTAGGGCAA
SS39	CCATTCCTCATCCAAACCAGGG
SS40	GAGCAGGGCTGAAAGACAGC
SS41	TTCCTGGTAGAATGTATGAAATGCG
SS42	ATCCCTGCTCCAGAGCACAG
SS43	ATGTGACCCTCCTGGCTG
SS44	GAAGCTATGGAAGGCCTTCATAGC
SS45	GGAAGCAGGGTCACACTCC
SS46	ACAGAAAGAGCACAGGACCAAC
SS47	TTGAAGCTTGGGATATCAGCATCTG
SS48	CCACTGCCACAGCAGACC
SS49	GCAGTCTCTGTAGCAGGGAG
SS50	GTGGTCAAAGAGTTGTGACTGGT
SS51	CCCTTCTGGGAGAGGGTCC
SS52	CCTAGCAGCCATCCCACAC
SS53	ATGGTAAGCTTTGCAGGCTG
SS54	TCTTCCTCAGTGGTCCCTCAC
SS55	CCAGCCGTGTCCTGAAGGA
SS56	ATTTCAGGACCACTAGAGAGCC
SS57	ACAACTGACATGCATCACTGTAC
SS58	CATGTAGAGTTATTGCTTGTCCTCC
SS59	CCACGATTAACCCACTCTTGTCG
SS60	GTGGGCAGGGACAGAGTCA
SS61	GGCTAGTTTCCAACCCAGAGC
SS62	CCTGGACCCATCGCTCTGA
*3C nested PCR*	
IL-10 SS04’	TGTGGGGCAGTTGGTCAGA
MK2 SS53’	CTTTGCAGGCTGGGGAGA

To further verify this chromatin link, we purified the 3C-PCR products and cloned them into the pJET vector. DNA sequencing confirmed the 3C ligation site (MboI), which was flanked by the *IL-10* promoter and the *MK2* enhancer on each side (Fig. 5C and D). Taken together, these data suggest that during CVB4 infection, the *MK2* enhancer was folded to the promoter region of *IL-10* gene, and together they formed a chromatin interaction to promote IL-10 expression. However, treatment with umifenovir can reverse the alteration in chromatin conformation induced by CVB4 infection, so as to inhibit the expression of IL-10. This is the first time we demonstrated that chromatin remodeling may play an important role in CVB4 infection.

## DISCUSSION

The repeated viral outbreaks highlight the urgent need for broad-spectrum antiviral drugs to combat the increasing diversity of viruses. Most antiviral drugs act upon the viral replication cycle, and their efficacy appears to be poor when they are used alone. Very few antivirals can modulate the host immune response. Umifenovir is a highly effective, broad-spectrum antiviral agent and is reported to have features of both a direct-acting antiviral (DAA) and a host-targeting antiviral (HTA) in some viral infections (16–19) and of an HTA in others (20, 21). This article first revealed the DAA and HTA effects of umifenovir on CVB4 infection. Many studies have reported an effect of umifenovir on one or several stages of the viral replication cycle, such as cell entry (attachment and internalization) and replication, through interactions with both membranes and with viral and/or cellular proteins. As previous studies have shown (4), the inhibition of virus replication by umifenovir is closely related to its ability to downregulate IL-10 production. In this study, we reconfirmed the critical role of IL-10 in viral infections using an *IL-10* knockout RAW264.7 cell model and further confirmed the inhibitory effects of CVB4 replication and IL-10 expression by umifenovir.

IL-10 is an inhibitory cytokine and a key factor in viral infections (22, 23). Aside from being produced by a variety of cell types, IL-10 targets a wide range of cells, resulting in a broad anti-inflammatory effect. As a result of its ability to modulate both the local cytokine microenvironment and antigen presentation, IL-10 prevents the efficient development of T cell responses (reviewed in reference 24). IL-10 can also block autophagy and allow viral persistence through a IL-10/STAT3 pathway (25, 26). Some viruses can reduce the antiviral immune response by the immune-evasion strategy of inhibiting dendritic cells and macrophages pathways through IL-10-dependent mechanisms (reviewed in reference 27). For decades, scientists have made various attempts to eliminate viral infections by regulating IL-10 (22, 23, 28). Studies have demonstrated that regulating *IL-10* and blocking the programmed cell death 1 (PD-1) pathway elicit potent antiviral effects (29). However, very few antiviral drugs are known to target IL-10. Díaz-Valdés et al. restored and enhanced the activity of hyporesponsive T cells using a synthetic IL-10 inhibitory peptide (30), which plays a therapeutic role in hepatitis C virus (HCV) infection. Additionally, as a major suppressor of the immune response and a key player in human disease, the regulation of IL-10 has great potential for the treatment of inflammatory and neurodegenerative diseases, infection, and even cancer (8, 9, 31, 32). Blocking IL-10 receptors in human breast cancer cells has been reported to improve the rate of response to chemotherapy (33). In the B-16 melanoma model, IL-10 and IL-35 synergistically promote T cell depletion in the tumor microenvironment (TME) through numerous mechanisms (34). IL-10 is widely expressed in various immune cells and has different effects on antitumor immunity under different circumstances. Hanna et al. (35) believed that combination strategies involving IL-10 regulation could improve the efficacy of immunotherapy for chronic lymphocytic leukemia (CLL) and other cancer types. Therefore, as a rare antiviral drug that regulates IL-10 expression, umifenovir has considerable value in clinical applications.

The epigenetic regulatory mechanism of umifenovir has not been reported. This article has elaborated the role of histone modification and chromatin remodeling in the antiviral activity of umifenovir.

Histone modification is an important component of epigenetics. Different repressive marker histones (such as H3K9me3 and H3K27me3) and active marker histones (such as H4K16ac and H3K9ac) regulate gene transcriptional activation or silencing by interacting with histone-modifying enzymes and transcription factors (36, 37) and collectively maintaining the balance of gene transcription (38). Once this balance is disrupted, the phenotype of cells is very likely to be altered, leading to the occurrence of various diseases. Several studies have suggested that histone modification of viral genome or host genes associated with viral infection plays an important role in the process of viral replication or latency (14, 15, 39–41).

The present study found that CVB4 could reduce the methylation level of the repressive histones H3K9me3 and H3K27me3 and increase the acetylation level of the active histone H3K9ac in the promoter region of the *IL-10* gene, thus promoting the expression of IL-10. Umifenovir can inhibit the alterations in the histone epigenotype caused by CVB4, thus inhibiting the expression of IL-10. Furthermore, we found that downregulation of JMJD3, UTX, KDM4B, and HDAC6 by umifenovir significantly contributed to this histone modification process after CVB4 infection. Cheng et al. found that HDAC6 can be recruited to the promoter region of the *IL-10* gene to promote the expression of IL-10. HDAC11 can be recruited to the same region simultaneously and form a complex with HDAC6 to negatively regulate the expression of IL-10 (42, 43). Conversely, Zhang et al. demonstrated that HDAC6 inhibitors could increase the expression of IL-10 and reverse the mechanical hypersensitivity induced by chemotherapy to treat chemotherapy-induced peripheral neuropathy (44). This may be attributed to the different cell types involved in IL-10 expression. Holla et al. showed that JMJD3, an H3K27 demethylase, can induce IL-10 expression in M2 macrophages during Mycobacterium infection (45). However, in NK cells, the application of the JMJD3/UTX inhibitor GSK-J4 reduced IL-10 levels (46). In summary, regulation of histone modification may be an important mechanism for the inhibition of IL-10 expression by umifenovir.

Another important finding of this study was that umifenovir can reduce the expression of IL-10 by regulating chromatin conformation in the promoter region of the *IL-10* gene. An increasing number of studies (47, 48) have shown that some long noncoding RNAs (lncRNAs), SWI/SNF complexes, and H1 linker histones can alter the chromatin conformation through a certain mechanism and promote or inhibit the “promoter-enhancer” chromatin interaction within topologically associated domains (TADs) (49, 50). This mechanism allows genes to be selectively activated or silenced to change the cell phenotype and determine cell and tissue specificity and cell fate. Briefly, chromatin conformation can precisely modulate every process of gene regulation, including gene replication, repair, and transcription. A recent report revealed the importance of chromatin remodeling in viral infection and antiviral processes (51).

Presently, research on chromatin remodeling of the *IL-10* gene has rarely been reported in the literature. The current study revealed that CVB4 infections could introduce chromatin conformational changes to the *IL-10* gene promoter region and distal enhancer of the *MK2* gene, thus forming a chromatin loop. *MK2* is an important gene in the p38-MAPK signaling pathway. It is located 36 kb downstream of the *IL-10* gene on mouse chromosome 1 and is strongly associated with the expression of IL-10. It has been suggested that MK2 is crucial for the induction of IL-10 *in vivo* in persistent murine cytomegalovirus (MCMV) infection and that deletion of *MK2* may lead to significant downregulation of IL-10 (52). The chromatin loop formed between the *MK2* and *IL-10* genes suggests that CVB4 infection causes a distant enhancer located on intron 8 of *MK2* to fold to the promoter of the *IL-10* gene, thereby forming chromatin interactions and promoting the expression of IL-10. However, treatment with umifenovir reduced IL-10 expression by reversing this alteration in chromatin conformation. For the first time, we report this chromatin remodeling as an antiviral mechanism to regulate IL-10 expression. However, further studies are required to gain a more in-depth understanding of this chromatin interaction. We speculate that this may be related to the recruitment of transcription factors or the mediation of noncoding RNA (11, 53).

In conclusion, based on the excellent antiviral activity of umifenovir demonstrated in previous studies (4), this study was the first to describe the epigenetic mechanism by which umifenovir regulates three-dimensional chromatin conformations and histone epigenotypes to reduce IL-10 expression and inhibit CVB4 infection, which has far-reaching implications for the redevelopment and application of umifenovir.

## MATERIALS AND METHODS

### Cells, viruses, and mouse strains.

HeLa cells and RAW264.7 macrophages (preserved by our laboratory) were cultured in Dulbecco modified Eagle medium (DMEM) (Biological Industries [BioInd]) containing 10% (vol/vol) fetal bovine serum (FBS) under standard conditions. The viral titers (tissue culture 50% infective doses [TCID_50_] per milliliter) were calculated according to the Reed and Muench method (54). Then CVB4 (preserved by our laboratory) was used to inoculate cells at a TCID_50_ of 106.15/100 mL. BALB/c mice 5 to 6 weeks of age (15 to 18 g) were purchased from the Academy of Military Medical sciences in Beijing, China, and raised in a specific-pathogen-free (SPF) environment. The study protocol was approved by the ethics review board of the First Hospital of Jilin University (approval no. 2019199). All of the procedures were performed in accordance with the relevant policies in China.

### Drugs and reagents.

Umifenovir (no. 110915; Qianjiang Hengshuo Chemical Industry Co. Ltd., Hubei, China) was the investigational drug. For cytological experiments, umifenovir was dissolved in dimethyl sulfoxide (DMSO), whose volume was less than 0.05% to avoid inhibitory effects on viral replication (20). All the primers and sequencing data in this study were completed by Kumei Biological Technology Co. Ltd. (Jilin, China).

### Construction and identification of *IL-10* knockout RAW264.7 cells.

The *IL-10* gene sequence information was obtained from GenBank. Six sgRNAs (KO1, TATTGTCTTCCCGGCTGTAC; KO2, GCCACATGCTCCTAGAGCTG; KO3, GAAAGTCTTCACCTGGCTGA; KO4, CTTGGGTTGCCAAGCCTTAT; KO5, CCTGGGGCATCACTTCTACC; and KO6, AGCTGAAGACCCTCAGGATG) were designed from the first two exons of *IL-10* in order to target knockout of the expression of IL-10. We constructed six *IL-10* knockout vectors by cloning six sgRNAs into the lenti-CRISPR gRNA vector from the Zhang lab (55), and the sgRNAs were inserted downstream of the U6 promoter in the vector using BsmBI. Six groups of plasmids were divided into two groups, KO1-KO2-KO3 and KO4-KO5-KO6, and packaged as lentiviruses in 293T cells. Then the RAW264.7 cells were transfected with *IL-10* knockout lentiviruses and selected by puromycin to obtain monoclonal cells. The successful knockout mutant I-C5# was confirmed by PCR and sequencing analysis. Finally, the levels of IL-10 in the RAW264.7/KO, RAW264.7/WT, and RAW264.7/NC cells were detected 24 h after CVB4 (1.4 × 10^6^ TCID_50_) infection by ELISA to confirm the success of the knockout process.

To examine the viability of the *IL-10* knockout RAW264.7 cells, RAW264.7/WT, RAW264.7/KO, and RAW264.7/NC cells were seeded into a 24-well plate at 1 × 10^4^ cells/well and cultured in DMEM with 10% FBS for 7 days. Meanwhile, we observed the cellular morphology using an inverted microscope (Nikon, Japan) every day, recorded the number of cells, and drew the cell growth curve to validate that knockout of *IL-10* had no effects on cell survival.

### Effect of IL-10 deficiency on viral replication.

To examine the influence of IL-10 deficiency on CVB4 replication, RAW264.7/WT, RAW264.7/KO, and RAW264.7/NC cells were seeded into a 24-well plate at 2 × 10^5^ cells/well with four replicate wells per group and cultured for 24 h to adhere. CVB4 (1.4 × 10^6^ TCID_50_) were then added and incubated for 2 h. After the viral supernatant was discarded, the adherent cells were washed twice with phosphate-buffered saline (PBS) and cultured in DMEM with 2% FBS for 7 days. During this time, the supernatants were collected daily under sterile conditions and stored at −20°C for subsequent viral titer measurements.

HeLa cells were seeded into a 96-well plate at 1 × 10^4^ cells/well, and the supernatant-KO, supernatant-NC, supernatant-WT and control supernatant of each day, which were diluted in a 10-fold dilution series, were added simultaneously. Each group had four repeated wells. After 48 h in culture, the cytopathic effect (CPE) was observed under a microscope, the viral titers were calculated (−logTCID_50_) (54), and the lethality rates of infected HeLa cells were assessed using the CCK-8 assay.

### Antiviral effect of umifenovir *in vitro*, *in vivo*, and *ex vivo*.

Myocardial cells were prepared from heart tissues of newborn BALB/c mice (1 to 2 days old) (56) and were cultured (4 × 10^6^ cells/mL) in a 96-well plate. HeLa cells in the logarithmic growth phase were seeded into a 96-well plate at 1 × 10^4^ cells/well. RAW264.7 cells were cultured in a 24-well plate at 2 × 10^5^ cells/well. BALB/c mice were used to prepare splenocytes according to the conventional method (57). The obtained splenocytes were suspended in a 24-well plate (1 × 10^7^ cells/well). Splenocytes, myocardial cells, and HeLa cells were infected with CVB4 solution (at 1.4 × 10^6^ TCID_50_, 2.8 × 10^3^ TCID_50_, and 2.8 × 10^3^ TCID_50_, respectively), and different doses of umifenovir (4, 8, and 12 μM) were added simultaneously. In the normal- and viral-control groups, the same volume of culture medium was added. The CCK-8 assay was applied to assess the viability of myocardial cells and HeLa cells using a CCK-8 kit (Sigma-Aldrich) after 48 h in culture. The IL-10 expression levels in supernatants of each group were measured by ELISA (Boster) and RT-PCR methods, and CVB4 loads were quantified by RT-PCR. The primers of IL-10 and CVB4 are as follows: IL-10, 5′-GACTGGCATGAGGATCAGCAG-3′ (forward [F]) and 5′-GTTAGCAGTATGTTGTCCAGCTGG-3′ (reverse [R]), and CVB4, 5′-GTTAGTAGTCCTCCGGCCCC-3′ (F) and 5′-CTATTCGACACCGG ATGGCCAA-3′ (R).

Twenty-four male BALB/c mice were randomly divided into 4 groups (*n* = 6), and three groups were injected intraperitoneally with 0.2 mL of CVB4 (2.8 × 10^3^ TCID_50_) for 3 consecutive days to establish a myocarditis model. For the normal-control group, saline was injected. Two of the three infected groups were then treated with umifenovir at 0.2 and 0.6 mmol kg^−1^ of body weight day^−1^, while the viral-control and the normal-control group received the same volume of saline. Umifenovir was administered in 0.2 mL of solution by gavage twice a day from the day before infection for 8 days, and the mice were sacrificed 7 days after infection. Mouse serum was collected to assess IL-10 levels by ELISA, and part of the myocardial tissue specimens were employed for RNA extraction (58) and RT-PCR was performed to measure CVB4 loads and *IL-10* mRNA levels. The remaining myocardial tissue samples were fixed in 10% formaldehyde for making paraffin sections and stained with H&E.

### Direct-acting and host-targeting antiviral mechanisms of umifenovir.

HeLa cells in the logarithmic growth phase were seeded into a 96-well plate at 1 × 10^4^ cells/well and cultured for 24 h to adhere. All cells were divided into three groups (I, direct killing effect of umifenovir on virus; II, antiviral effect of umifenovir postadsorption; and III, protective effect of umifenovir). Each group was classified into 5 subgroups (viral control, 4 μM umifenovir, 8 μM umifenovir, 12 μM umifenovir, and normal control). In group I, CVB4 (2.8 × 10^3^ TCID_50_) and umifenovir (4, 6, or 12 μM) were mixed in a centrifuge tube, placed at 37°C for 2 h, and then added to cells in 96-well plates to incubate for 2 h; the cells were washed twice with PBS and added to culture medium containing umifenovir (4, 6, or 12 μM). In group II, CVB4 (2.8 × 10^3^ TCID_50_) was added to HeLa cells for 2 h and the cells were washed twice and then added to medium with umifenovir (4, 6, or 12 μM). In group III, umifenovir (4, 6, or 12 μM) was added to the cells for 2 h, the cells were washed twice, then CVB4 (2.8 × 10^3^ TCID_50_) was added for 2 h, and the cells were washed twice again and then added to medium with umifenovir (4, 6, or 12 μM). After treatment, the three groups of cells were cultured for another 48 h, and the cytopathic effect (CPE) was observed under the microscope. The survival rate of HeLa cells was detected with the CCK-8 kit to explore the antiviral mechanism of the drug.

### Histone ChIP assay.

Splenocytes were obtained from BALB/c mice according to the conventional method (57) and were suspended in a 24-well plate (1 × 10^7^ cells/well). All mouse splenocytes were divided into three groups (normal control, viral control, and umifenovir treatment group). The viral control and umifenovir treatment group were infected with CVB4 (1.4 × 10^6^ TCID_50_), and the umifenovir treatment group was treated with umifenovir (12 μM) additionally. Then the three groups of splenocytes were collected after 48 h in culture for the subsequent chromatin immunoprecipitation (ChIP) experiment.

ChIP assays were performed using a ChIP kit (Abcam, MA) according to the manufacturer’s protocol. Three groups of splenocytes (1 × 10^6^ cells/group) were collected and fixed with 1% formaldehyde for 10 min at room temperature. Then SDS nuclear lysis, ultrasonic DNA shearing, protein and DNA immunoprecipitation, cross-linked DNA reversal, and DNA purification were conducted step by step. Anti-H3K9me3, -H3K9ac, and -H3K27me3 antibodies (Abcam) were used for coimmunoprecipitation, and mouse IgG (Abcam) was used for the negative control. The purified coprecipitated DNA fragments were used to detect fragments from IL-10 promoter (F, 5′-GAGACGTGTAACCTGTAGCT-3′, and R, 5′-GTAGGAGAAGTCCCTACTGA-3′) by quantitative real-time PCR (RT-qPCR).

### Quantitation of gene expression by qPCR.

RT-qPCR was performed using the 2× RealStar green fast mixture (with ROX reference dye) (GenStar, Beijing, China) with a Bio-Rad CFX384 Touch real-time PCR detection system (Bio-Rad, Hercules, CA). PCR cycle conditions were as follows: 1 cycle at 95°C for 2 min, 35 cycles at 95°C for 15 s, 65°C for 30 s, and 72°C for 15 s, and 1 cycle at 65°C for 10 min. For quantitative real-time PCR, the threshold cycle (*C_T_*) values of target genes were normalized over the *C_T_* of the glyceraldehyde-3-phosphate dehydrogenase (GAPDH) control (GAPDH primers: F, 5′-AGGAGTAAGAAACCCTGGAC-3′, and R, 5′-CTGGGATGGAAATTGTGAG-3′).

### mRNA levels of the histone-modifying enzyme.

To examine the influence of umifenovir on the mRNA levels of histone-modifying enzyme in CVB4 infection, five groups (normal control, viral control, and 4, 8, and 12 μM umifenovir treatment groups) of mouse splenocytes as described previously were collected and used for total RNA extraction. Concentration and quality of all RNA samples were evaluated by NanoDrop 1000 (Thermo Scientific, CA), and the 260/280 and 260/230 values of all samples were between 1.8 and 2.0. The extracted RNA samples were stored at −80°C for cDNA synthesis using StarScript II first-strand cDNA synthesis mix with DNA remover (GenStar). The sequences of PCR primers were as follows: JMJD3, 5′-CTGCTGTAACCCACTGCTGGA-3′ (F) and 5′-GAAAGCCAATCATCACCCTTGTC-3′ (R); UTX, 5′-ATCCCAGCTCAGCAGAAGTT-3′ (F) and 5′-GGAGGAAAGAAAGCATCACG-3′ (R); KDM4B, 5′-TGGCCTGGCCAAGATCATT-3′ (F) and 5′-CCACAGTCATGGCCTTCTTCTG-3′ (R); and HDAC6, 5′-AGTAGCCAAGTAGGCCGAGA-3′ (F) and 5′-GTGAGGTGGGCATAACCCTC-3′ (R).

### 3C.

Three groups (normal control, viral control, and 12 μM umifenovir treatment group) of mouse splenocytes as described for the ChIP method were collected after 48 h in culture. As previously reported (59), after being washed twice with PBS, cells (1 × 10^6^/group) were cross-linked by 2% formaldehyde at room temperature for 10 min. Then, the cells were lysed with lysis buffer, and the fixed DNA was sheared with MboI restriction endonuclease (R0147; New England BioLabs [NEB]) at 37°C for at least 2 h. T4 DNA ligase (M0202; NEB) was used to religate DNA fragments. After ligation, proteinase K was used to reverse the cross-links, and DNAs were then purified for subsequent PCRs. Primers of the *IL-10* promoter and *MK2* gene were paired up sequentially, and the nested PCR method was performed to obtain chromosome conformation capture PCR (3C-PCR) products. The primers for 3C-PCR were designed in regions of *IL-10* promoter and the whole *MK2* gene (Table 1). The 3C-PCR products were then purified and inserted into a pJET vector (Thermo, CA) for sequencing.

### Statistical analysis.

All experiments were performed in quadruplicates. The data were expressed as means ± standard errors of the means (SEM) and were analyzed using SPSS software (version 20.0.0). The data were analyzed with Student’s *t* test or by one-way analysis of variance, and statistically significant differences were determined by Student’s *t* test.
